# A Positive Feedback Synapse from Retinal Horizontal Cells to Cone Photoreceptors

**DOI:** 10.1371/journal.pbio.1001057

**Published:** 2011-05-03

**Authors:** Skyler L. Jackman, Norbert Babai, James J. Chambers, Wallace B. Thoreson, Richard H. Kramer

**Affiliations:** 1 Department of Physics, University of California, Berkeley, Berkeley, California, United States of America; 2 Department of Ophthalmology, University of Nebraska Medical Center, Omaha, Nebraska, United States of America; 3 Department of Chemistry, University of Massachusetts, Amherst, Amherst, Massachusetts, United States of America; 4 Department of Molecular and Cell Biology, University of California, Berkeley, Berkeley, California, United States of America; University of Washington, United States of America

## Abstract

Cone photoreceptors and horizontal cells (HCs) have a reciprocal synapse that underlies lateral inhibition and establishes the antagonistic center-surround organization of the visual system. Cones transmit to HCs through an excitatory synapse and HCs feed back to cones through an inhibitory synapse. Here we report that HCs also transmit to cone terminals a positive feedback signal that elevates intracellular Ca^2+^ and accelerates neurotransmitter release. Positive and negative feedback are both initiated by AMPA receptors on HCs, but positive feedback appears to be mediated by a change in HC Ca^2+^, whereas negative feedback is mediated by a change in HC membrane potential. Local uncaging of AMPA receptor agonists suggests that positive feedback is spatially constrained to active HC-cone synapses, whereas the negative feedback signal spreads through HCs to affect release from surrounding cones. By locally offsetting the effects of negative feedback, positive feedback may amplify photoreceptor synaptic release without sacrificing HC-mediated contrast enhancement.

## Author Summary

Visual images are projected by the lens of the eye onto a sheet of photoreceptor cells in the retina called rods and cones. Like the pixels in a digital camera, each photoreceptor generates an electrical response proportional to the local light intensity. Each photoreceptor then initiates a chemical signal that is transmitted to downstream neurons, ultimately reaching the brain. But unlike the pixels of a digital camera, photoreceptors indirectly inhibit one another through laterally projecting horizontal cells. Horizontal cells integrate signals from many photoreceptors and provide inhibitory feedback. This feedback is thought to underlie “lateral inhibition,” a process that sharpens our perception of contrast and color. Here we report the surprising finding that horizontal cells also provide positive feedback to photoreceptors, utilizing a mechanism distinct from negative feedback. The positive feedback signal is constrained to individual horizontal cell–photoreceptor connections, whereas the negative feedback signal spreads throughout a horizontal cell to affect many surrounding photoreceptors. By locally offsetting negative feedback, positive feedback boosts the photoreceptor signal while preserving contrast enhancement.

## Introduction

The retina is an exceptionally approachable part of the brain, hence deciphering the retinal neural circuit was one of the earliest triumphs of systems neuroscience [1]. The basic wiring diagram of the retina was determined largely from electrical recordings from each of the main neuronal cell types. Synaptic connections were first deduced by examining how the light response is transformed from one retinal cell type to the next [2]. Paired recordings from different cell types and anatomical and pharmacological studies confirmed the occurrence of these connections and helped define their functional properties.

The synaptic connection between HCs and cone photoreceptors attracted particular interest right from the beginning [3],[4]. Voltage changes in HCs result in sign-inverted voltage changes in cone photoreceptors, a negative feedback connection. HCs project laterally in the retina over hundreds of microns and integrate inputs from many rods and cones, so negative feedback causes cones [3] and rods [5] to have an antagonistic center-surround receptive field. This receptive field organization is reflected postsynaptically first in bipolar cells [2] and in subsequent neuronal layers of the visual system [6], enhancing the neural representation of spatial contrast and sharpening visual detection of edges.

Despite decades of study, the mechanism of negative feedback from HCs remains controversial. Three main hypotheses have been advanced to explain how this sign-inverting synapse works; that is, how depolarization of the HC inhibits neurotransmitter release from cones. First, it was proposed that HCs release the neurotransmitter GABA, hyperpolarizing the cone membrane potential [7]. Second, an ephaptic mechanism was proposed, in which electrical current through channels in HC dendrites locally changes the transmembrane potential of the cone terminal [8],[9]. The ephaptic signal is proposed to mediate negative feedback and modulate the gain of the cone synapse [10]. Third, it was proposed that depolarization of HCs causes the efflux of protons, which acidifies the extracellular space and inhibits cone voltage-gated Ca^2+^ channels [11],[12]. The debate continues over which of these mechanisms predominate in generating negative feedback to cones.

Here we report the surprising discovery that HCs also generate *positive* feedback onto cones, distinct from the negative feedback signal that has been studied for the past 40 years. Optical imaging methods reveal that the cone neurotransmitter glutamate triggers a retrograde signal from HCs, which elevates intracellular Ca^2+^ in cones and enhances neurotransmitter release. This signaling system is robust in the intact retina but disrupted in retinal slices, which are often used for investigating the HC synapse. We propose that the positive feedback synapse between HCs and photoreceptors locally offsets the effect of negative feedback and boosts photoreceptor transmission, preserving signal strength in the visual system without sacrificing HC-mediated contrast enhancement.

## Results

### Glutamate Increases Synaptic Release from Cone Terminals

**Figure 1:**
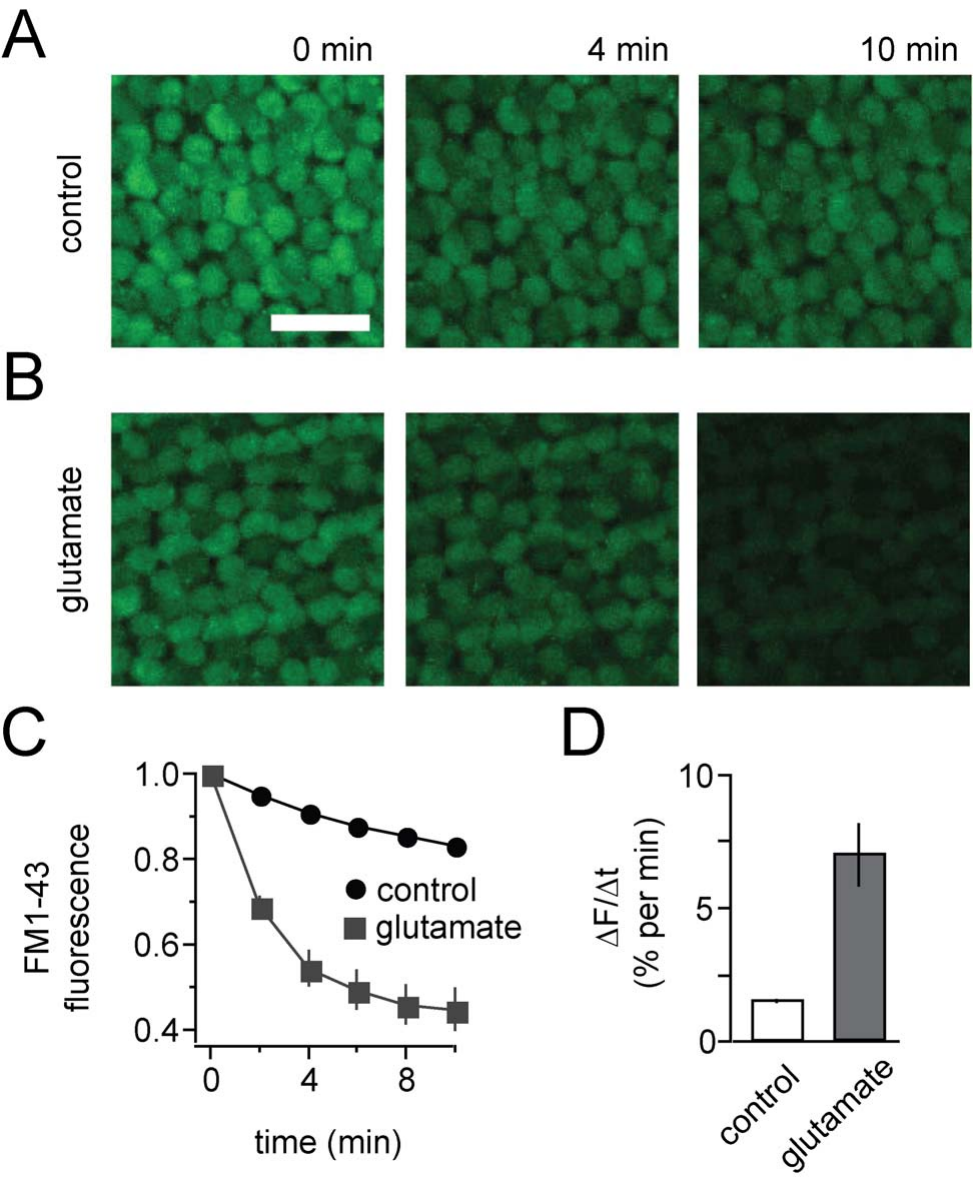
Glutamate accelerates synaptic release from cone terminals. (A) Fluorescence images of cone terminals in the outer plexiform layer of a flat-mounted anole retina loaded with FM1-43. Terminals continuously release FM1-43 in darkness as a result of tonic vesicle release. Scale bar  =  20 µm. (B) Addition of 2 mM glutamate to the bath solution accelerates release from cone terminals. (C) Time-course of FM1-43 fluorescence decreases from cone terminals in darkness (*n* = 27, error bars are obscured by the data points) and 2 mM glutamate (*n* = 5). (D) Average rates of FM1-43 release (ΔF/Δt). Release in glutamate is 4-fold faster than the rate in darkness (control). Data in this and subsequent figures are expressed as mean ± SEM.

To investigate feedback at the cone-HC synapse we monitored synaptic vesicle release from cone terminals with fluorescence microscopy. As previously described, the all-cone retina of the anole lizard was dark-adapted in physiological saline containing the amphipathic dye FM1-43 [13]. In darkness, cone terminals support continuous exocytosis and compensatory endocytosis. Vesicles internalized during endocytosis incorporate the dye, producing brightly labeled cone synaptic terminals. Washing the retina with a solution containing Advasep-7 removes FM1-43 from the surface membranes of cells but spares the dye in internalized vesicles. Subsequent loss of dye from synapses results from the exocytosis of labeled vesicles [13],[14], and this can be monitored with an infrared 2-photon laser-scanning microscope (Figure 1A).

To elicit feedback from HCs onto cones we added glutamate to the bath solution. HCs depolarize when glutamate activates ionotropic glutamate receptors on their dendrites [15],[16]. Depolarized HCs feed back onto cones by inhibiting the voltage-gated Ca^2+^ channels that support exocytosis [11],[17],[18]. Hence, the predicted effect of HC depolarization is a *decrease* in the rate of synaptic release from cones. Remarkably, the addition of glutamate *increased* release from cone terminals (Figure 1B), with the rate of exocytosis increasing ∼4-fold over the rate in darkness (Figure 1C).

### Ionotropic Glutamate Receptors Are Responsible for Accelerating Cone Release

**Figure 2:**
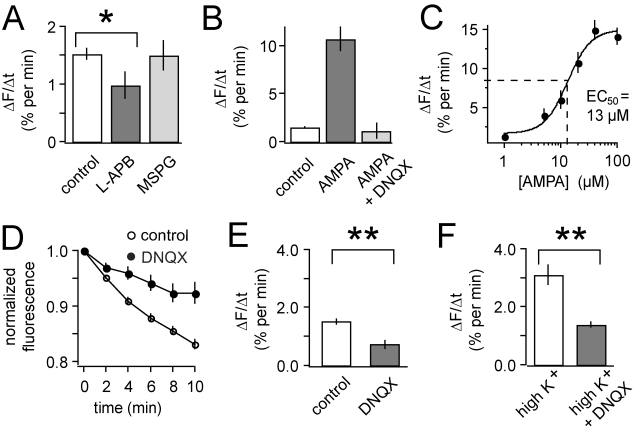
AMPA receptors mediate the acceleration of cone synaptic release. (A) Bath addition of 20 µM L-APB (an mGluR group III agonist) significantly slows release from cones in darkness (*p*<0.05, *n* = 13). The mGluR antagonist MSPG (100 µM) has no effect on the dark release rate. (B) 20 µM AMPA markedly increases the release rate from cones (*n* = 16). The effect of AMPA is blocked by 10 µM DNQX (*n* = 10), leaving release insignificantly changed from the control rate in darkness. (C) Dose-response curve for release rate as a function of AMPA concentration. *N* = 4–16 for each concentration. (D–E) Bath addition of 10 µM DNQX significantly slows release from cone terminals in darkness (*p*<0.001, *n* = 21), suggesting that the physiological level of ambient glutamate boosts release. (F). High K^+^ (50 mM) saline, which depolarizes cones, accelerated release from cone terminals to twice the dark release rate (*n* = 9). Addition of 10 µM DNQX greatly reduced the high K^+^ evoked-release (*p*<0.01, *n* = 7), suggesting that the glutamate released by cones elicits positive feedback. In this and subsequent figures, * denotes statistical significance of *p*<0.05, while ** denotes *p*<0.01.

Glutamate activates ionotropic receptors (iGluRs), metabotropic receptors (mGluRs), and plasma membrane transporters. To ascertain which of these is responsible for accelerating release from cones, we started by applying selective agonists and antagonists of mGluRs. Cones are known to possess group III mGluRs that can regulate intraterminal Ca^2+^ [19], but mGluR agonists decrease synaptic release [20]. In agreement with this, we found the mGluR group III-selective agonist L-APB slowed FM1-43 release from dark-adapted cones (Figure 2A). Blocking cone mGluRs with the group II/III antagonist MSPG failed to increase release, indicating that cone mGluRs are not tonically activated in darkness, when glutamate release is high.

Glutamate also binds to and activates plasma membrane transporters in cones, triggering a Cl^−^ current [21]. The current is usually hyperpolarizing, but under some conditions it might depolarize the cone and activate voltage-gated Ca^2+^ channels, increasing the release rate. To preclude activation of transporters, we used the iGluR-selective agonist AMPA, which depolarizes HCs but does not affect the glutamate transporter [22] and has no detectable direct action on photoreceptors [15],[19]. Similar to glutamate, AMPA caused a large (∼6-fold) increase in the release rate from cone terminals (Figure 2B). The AMPA/kainate receptor antagonist DNQX blocked the effect of AMPA. A dose-response curve for AMPA reveals an EC_50_ of 13 µM (Figure 2C), similar to the EC_50_ of 15 µM for AMPA receptors in isolated catfish HCs [15].

Taken together, these results establish that iGluRs are responsible for triggering the increase in vesicular release from cone terminals. In contrast to mGluRs and glutamate transporters, which might play a negative feedback role in regulating cone release, iGluRs play a positive feedback role in augmenting the release rate. Cone terminals do not appear to possess iGluRs [23],[24], implying that AMPA-induced acceleration of cone release operates through a multi-cellular pathway, for example involving HCs.

To test whether there is sufficient glutamate released in darkness to activate the positive feedback mechanism, we applied DNQX. DNQX significantly decreased the FM1-43 release rate by 52%±14% (*p*<0.001, Figure 2D, 2E). Hence the ambient concentration of glutamate at the synapse in darkness is sufficient to activate the positive feedback system.

Depolarizing the cone beyond the dark membrane potential elicits more glutamate release, which could further increase positive feedback. High K^+^ (50 mM) saline evoked a 2-fold increase in exocytosis as compared to darkness (Figure 2F), and blocking AMPA receptors with DNQX significantly reduced high K^+^-elicited release by 56%±12% (*p*<0.01). The observation that DNQX reduces cone release both in darkness and in high K^+^ suggests that positive feedback operates over much of the dynamic range of the cone synapse, helping to set the physiological release rate.

If elevated glutamate at the synapse can trigger positive feedback, suppressing glutamate removal from the synapse should enhance cone release. Consistent with this prediction, we found that TBOA, an inhibitor of the plasma membrane glutamate transporter [21], increases cone release in darkness by 45%±9% (*p*<0.005). However, TBOA does not reduce AMPA- or glutamate-accelerated release, confirming that the glutamate transporter is not required for positive feedback.

We found that AMPA increased vesicular release from retinal photoreceptors in species across several phyla, including zebrafish (*Danio rerio*), tiger salamander (*Ambystoma tigrinum*), anole lizard (*Anolis carolinensis*), and rabbit (*Oryctolagus cuniculus*) (Figure S1). In each species AMPA increased the release rate by >2-fold as compared to darkness. The enhancement of release was uniform over the variety of cone and rod terminals found in the outer plexiform layer of these retinas, indicating that AMPA augments release from both rods and cones. In the rod-only retina of the gecko (*Gecko gecko*) AMPA also increases release from rods. In anole retina, AMPA increased the release rate to about the same final value whether the retina was dark-adapted or light-adapted (Figure S2), suggesting that AMPA receptor-regulated release operates through a mechanism that is distinct from light-regulated release.

### Narrowing Down the Source of the Positive Feedback Signal

**Figure 3:**
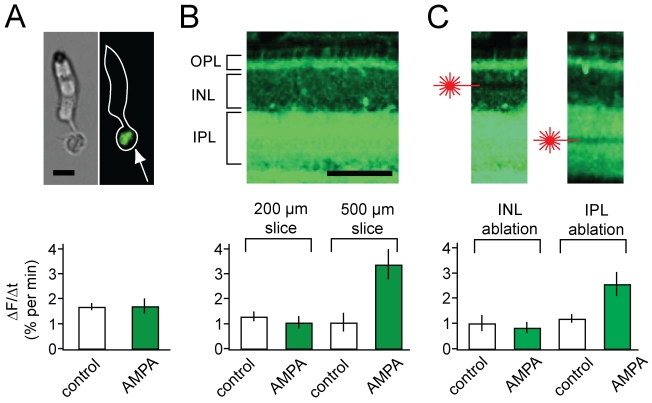
Determining the cellular locus of AMPA-accelerated release. (A) AMPA has no effect on release from isolated cones. Top: Images of a dissociated cone photoreceptor from a FM1-43 loaded anole retina; brightfield (left), fluorescence (center), and overlay of both images (right). Dye fluorescence is localized to the synaptic terminal (arrow). Scale bar  =  10 µm. Bottom: Release rate from cones in 20 µM AMPA (*n* = 6) is the same as in control cones without AMPA (*n* = 6). (B) AMPA accelerates release in thick, but not thin, retinal slices. Top: Fluorescence image of a retinal slice, loaded with FM1-43 prior to slicing. Bottom: 20 µM AMPA has no effect on release in thin (200 µm) slices (*n* = 9) but accelerates release by ∼2.5-fold in thick (500 µm) slices (*n* = 10). (C) Specific laser ablation of the HC layer disrupts AMPA-accelerated release. Top: Fluorescence image of a 500 µm-thick slice after laser ablation of either the region of the INL containing HC bodies (left) or the center of the IPL containing processes of other retinal neurons (right). Bottom: Ablation of the INL (*n* = 11), but not the IPL (*n* = 6), disrupts AMPA-induced release. Scale bar  =  100 µm in (A) and (B). Laser-ablated regions extend 600 µm laterally along the surface of the slice.

Our results indicate that iGluRs are responsible for augmenting cone release, yet studies suggest that cone photoreceptors do not possess iGluRs [23],[24]. To confirm that functional AMPA receptors are absent from cones, we examined release from cones acutely isolated from the retina. Retinas loaded with FM1-43 were treated with papain and mechanically triturated to isolate individual cones. The dissociated cones retained bright FM1-43 fluorescence at their terminals (Figure 3A, top) and spontaneously released the dye at a rate similar to that measured in the intact retina. As expected, AMPA had no effect on release from dissociated cones (Figure 3A, bottom).

We next tested the effect of AMPA on retinal slices. The transverse retinal slice is a popular preparation for studying the synapse between HCs and photoreceptors because it provides unimpeded access for patch-clamp recordings. However, slicing can damage HCs, whose processes extend laterally over hundreds of microns, and thus might compromise HC feedback. Indeed, when we prepared 200- µm-thick slices from FM1-43 loaded retinas, AMPA failed to accelerate release from cones. When we prepared larger 500 µm-width slices, AMPA could still accelerate release from cones but only half as much as in the flat-mounted retina (Figure 3). Because the width of these slices should not affect the health of the cones whose diameter is ∼10 µm, these results suggest that AMPA-induced feedback operates through cells that project over a more extended region (>200 µm).

To further investigate the source of positive feedback to cones, we used laser ablation to disrupt various neuronal layers in 500- µm-thick slices of the anole retina. Prior to AMPA application, the power of the imaging laser was increased from ∼20 mW to ∼2 W. We scanned along the slice to induce cell damage in either the portion of the inner nuclear layer (INL) where HC somata reside or in the inner plexiform layer (IPL), which contains processes of amacrine, bipolar, ganglion, and interplexiform cells (IPCs), but not HCs (Figure 3C, top). Laser ablation produced immediate cell damage, which was apparent from cellular blebbing and the loss of dye in the scanned region. There was no significant difference in the dark release rate from cone terminals caused by ablation of either the INL or IPL (Figure 3C, bottom). When the laser was targeted to the INL to ablate HCs, AMPA failed to accelerate FM1-43 release from cone terminals. However, when the laser was targeted to the IPL, there was no significant difference in the effect of AMPA on cone release from slices with and without laser ablation (Figure 3B,C, *p* = 0.41). These results implicate cells with processes in the INL, but not in the IPL, as the source of positive feedback.

HCs and IPCs are the only two laterally projecting neurons in the retina that are known to contact cones. There are several different types of IPCs containing different neurotransmitters including dopamine [25] and glycine [26] and receptors for these transmitters are found on cone terminals [27],[28]. To ascertain whether IPCs might be the source of positive feedback onto cones, we asked whether AMPA could still accelerate the cone release rate after applying agonists or antagonists of dopamine or glycine receptors. AMPA acceleration of cone release was unaffected by dopamine (100 µM) or glycine (1 mM), and the glycine receptor antagonist strychnine (1 µM) also failed to block AMPA-accelerated release (Figure S3). Hence it seems unlikely that IPCs are the source of AMPA-elicited positive feedback, focusing our attention on HCs.

### Putative Negative Feedback Mechanisms Are Not Involved in Positive Feedback

Three mechanisms have been proposed to account for negative feedback regulation of cone neurotransmitter release by HCs: (1) GABA-ergic feedback, (2) electrical (ephaptic) feedback, and (3) proton-mediated feedback. To evaluate whether any of these mechanisms is involved in AMPA-elicited positive feedback, we manipulated each of these systems with pharmacological agents while monitoring FM1-43 release from flat-mounted anole retinas.

**Figure 4:**
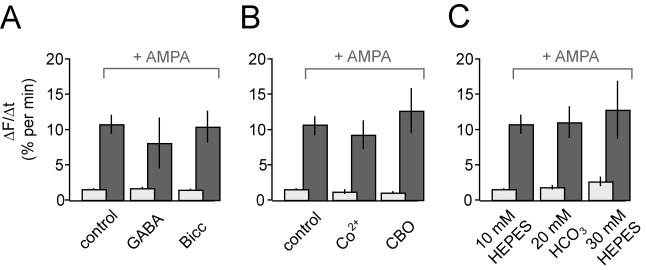
Blockers of hypothesized mechanisms of negative feedback have no effect on AMPA-accelerated release from cone terminals. (A) Tonic release in darkness is unaffected by GABA (500 µM; *n* = 8) or bicuculline (100 µM; *n* = 5) and AMPA-accelerated release is not significantly altered by either GABA (*n* = 6, *p* = 0.15) or bicuculline (*n* = 5, *p* = 0.56). (B) Tonic release is slightly reduced by the hemichannel blockers Co^2+^ (100 µM; *n* = 8) and carbenoxolone (CBO; 100 µM; *n* = 10), but neither Co^2+^ (*n* = 8) nor CBO (*n* = 8) blocks AMPA-accelerated release. (C) Tonic release is slightly increased by increasing the pH buffer concentration from 10 to 30 mM HEPES (*n* = 8) but unaffected by substituting HEPES with the weaker pH buffer HCO_3_ (20 mM) (*n* = 7). However, neither 30 mM HEPES (*n* = 6) nor HCO_3_ (*n* = 7) blocks AMPA-accelerated release.

For many years, GABA was the leading candidate as the HC negative feedback signal [7],[29]. In this scenario, GABA released from HCs activates GABA_A_ receptors on cone terminals, hyperpolarizing the cone membrane potential and suppressing neurotransmitter release. However, GABA_A_ antagonists do not block negative feedback [30],[31] and rather than regulating a Cl^−^ conductance in cones as predicted by the GABA hypothesis, HC feedback appears to regulate a voltage-gated Ca^2+^ conductance in cones [18],[32]. These and other studies challenge the role of GABA as the mediator of negative feedback, but we considered the possibility that GABA could be involved in positive feedback. We found that neither GABA nor bicuculline, a GABA_A_ antagonist, had a significant effect on the rate of FM1-43 release from cone terminals (Figure 4A). Moreover, applying GABA or bicuculline for 20 min prior to AMPA did not significantly change the AMPA-induced increase in FM1-43 release. These results suggest that GABA is not the positive feedback signal.

The second hypothesis is that negative feedback from HCs is electrical in nature. This “ephaptic” hypothesis states that electrical current through ion channels in the tips of HC dendrites causes a local change in the extracellular voltage, shifting the activation curve in cones such that a larger depolarization is needed to activate voltage-gated Ca^2+^ channels [8],[9]. HCs possess connexin hemichannels, and HC-mediated negative feedback can be blocked with the hemichannel blockers Co^2+^ [30] or carbenoxolone (CBO) [9]. We used these blockers to test the possible involvement of hemichannels in AMPA-elicited positive feedback. Both Co^2+^ and CBO caused a small but significant decrease in the dark rate of release (Figure 4B). Both reagents reportedly inhibit cone voltage-gated Ca^2+^ channels [33],[34], which could explain the decreased release rate, although the concentration of Co^2+^ used (100 µM) should have a minimal effect on photoreceptor voltage-gated Ca^2+^ channels [30]. More to the point, neither Co^2+^ nor CBO blocked the AMPA-induced increase in release. These results rule out hemichannels as mediating AMPA-elicited positive feedback.

The third hypothesis is that depolarization of HCs leads to the extrusion of protons through pumps or channels, acidifying the extracellular space and inhibiting the activation of voltage-gated Ca^2+^ channels in the cone terminal. Supporting this hypothesis, HC-mediated negative feedback is blocked by high concentrations of strong pH buffers [11],[12],[35]. To test whether protons play a role in AMPA-elicited positive feedback, we performed similar experiments, comparing the effect of AMPA on cone release with bath solutions that contained either HEPES, a strong buffer that blocks negative feedback, or HCO_3_ (bicarbonate), a weak buffer that preserves negative feedback. We found that a high concentration of HEPES slightly increased the release rate of cones in darkness as compared to HCO_3_ (Figure 4C), consistent with inhibition of negative feedback. However, the AMPA-elicited increase in release was the same in HCO_3_ and HEPES, inconsistent with positive feedback being mediated by a change in pH.

### Glutamate Receptor Activation Triggers an Increase in Ca^2+^ in Cone Terminals

**Figure 5:**
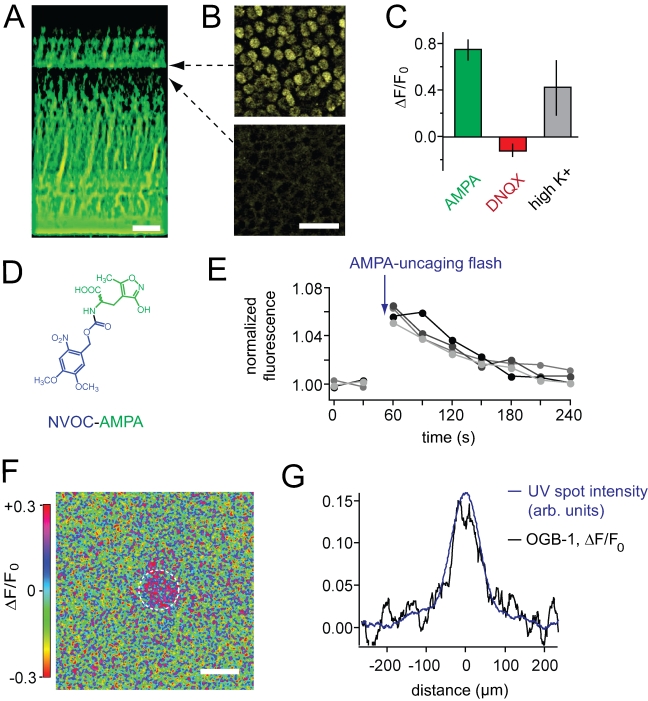
AMPA triggers a rise in Ca^2+^ in the cone synaptic terminal. Incubating anole retina with 100 µM of the AM-ester form of Oregon Green BAPTA-1 (OGB) resulted in robust loading of Ca^2+^ indicator dye into cone terminals. (A) A “virtual slice” of a flat-mounted retina loaded with OGB, rendered using a z-stack of 80 cross-sectional images. Images were taken at 2 µm intervals through the entire retina using a 2-photon microscope. Labeling is selective for photoreceptors (top) and retinal ganglion cells and Muller glial cells (bottom). (B) Representative cross-sectional images of OGB fluorescence in a flat-mounted retina. Cone terminals (top) are brightly labeled, while the region of the INL immediately below the cone terminals is not (scale bar  =  20 µm, A,B). (C) Average peak OGB-1 fluorescence change caused by AMPA (*n* = 5), DNQX (*n* = 4), and high K^+^ (*n* = 5). (D) NVOC-AMPA, a photolyzable agonist for selective optical activation of AMPA receptors. (E) Four uncaging trials from an OGB-1-loaded retina show that uncaging NVOC-AMPA (50 µM) with UV light triggers rapid, reversible increases in cone terminal Ca^2+^. (F) OGB-1 fluorescence increase in a flat-mounted retina triggered by flash uncaging of NVOC-AMPA. The dotted circle represents the full-width half-maximal of the Guassian region of uncaging. Scale bar =  100 µm. (G) Profile of the OGB-1 fluorescence increase in a flat-mounted retina and of the UV uncaging spot.

Neurotransmitter release from cones is Ca^2+^-dependent, so we next asked whether AMPA leads to a rise in intracellular Ca^2+^ in the cone terminal. Previous studies showed that iGluR agonists fail to elevate cone terminal Ca^2+^ in retinal slices [12],[36], but we know that positive feedback is impaired in the slice preparation. Flat-mounted anole retinas were incubated with the AM-ester form of Oregon Green BAPTA-1 (OGB-1), resulting in incorporation of Ca^2+^ indicator dye into cone photoreceptors, retinal ganglion cells, and Muller cells (Figure 5A). Robust OGB-1 labeling was seen in cone terminals, but not in the adjacent horizontal cells (Figure 5B). Application of AMPA triggered a large increase of Ca^2+^ (Figure 5C). In contrast, DNQX caused a small but significant (*p*<0.05) decrease in Ca^2+^ (Figure 5C). This result indicates that the ambient activation of AMPA receptors is sufficient to keep intracellular Ca^2+^ elevated, again suggesting that the positive feedback system is operating in darkness. High K^+^ saline also elevated intracellular Ca^2+^ (Figure 5C), but to a lesser extent than AMPA. This agrees with the results of Figure 2, which show that FM1-43 release from cone terminals is accelerated more by AMPA than by high K^+^.

AMPA triggered a persistent rise in intracellular Ca^2+^ in cone terminals that was difficult to reverse, even with prolonged washing. To confirm that the rise in Ca^2+^ is reversible, we needed a faster and more precisely targeted method for activating AMPA receptors. A particularly powerful approach involves the photolysis of a caged neurotransmitter agonist, for example 4-methoxy-7-nitroindolinyl (MNI)-glutamate [37]. However, glutamate acts on many receptor types in the retina, and while iGluRs and mGluRs can be blocked selectively, blockade of glutamate transporters will lead to an increase in the ambient level of glutamate, confounding our results. In fact, uncaging of MNI-glutamate triggered oscillations of Ca^2+^ in cone terminals that may have been caused by activation of glutamate transporters (unpublished data).

To circumvent this problem, we chemically synthesized a form of caged AMPA, which upon photolysis should activate AMPA receptors but not glutamate transporters. We synthesized the nitroveratryl carbamate derivative of AMPA (NVOC-AMPA) (Figure 5D), which contains the photolabile NVOC protecting group that can be removed with exposure to 365 nm light. Using NVOC-AMPA on the OGB-loaded anole retina, we found that flashes of UV light could trigger a repeated transient rise in Ca^2+^ in the cone terminals (Figure 5E). Unlike glutamate, AMPA is not removed from the extracellular space by plasma membrane glutamate transporters, which may account for the slow decay of these responses. Our ability to resolve the time-course of the cone Ca^2+^ increase was hindered by the necessity that we change optical elements in the microscope when switching from two-photon imaging to UV-uncaging. However, our experiments show that Ca^2+^ peaks within 5 s of the end of the uncaging flash, the shortest interval we could achieve. The UV uncaging flashes had no measurable effect on cone Ca^2+^ when the caged molecule was not present. These experiments demonstrate that AMPA receptor activation causes a rapid, reversible rise in Ca^2+^ in cone terminals.

To evaluate the spatial spread of the positive feedback signal, we uncaged AMPA in a small circular region of the OPL (100 µm diameter) and measured the resulting increase in cone terminal Ca^2+^ (Figure 5F). We found that the spatial profile of Ca^2+^ elevation in the underlying array of cone terminals closely matched the area of AMPA uncaging (Figure 5G). Hence positive feedback appears to remain tightly localized to the AMPA-activated region, in contrast to negative feedback, which can spread widely not only within an individual HC but between coupled networks of HCs connected through gap junctions [1],[38].

### The Positive Feedback Signal Activates a Voltage-Independent Conductance in Cones

We next turned to electrophysiology to compare positive and negative feedback. Previous patch clamp studies showed that depolarization of HCs leads to inhibition of voltage-gated Ca^2+^ channels in cones [18], a key consequence of negative feedback. We confirmed this effect by recording from synaptically connected HCs and cones in a retinal slice. We used the retina from tiger salamander because the compact structure of their cones allows for more effective voltage-clamp of the synaptic terminal than lizard cones, which have a long axon separating the terminal from the cell body.

**Figure 6:**
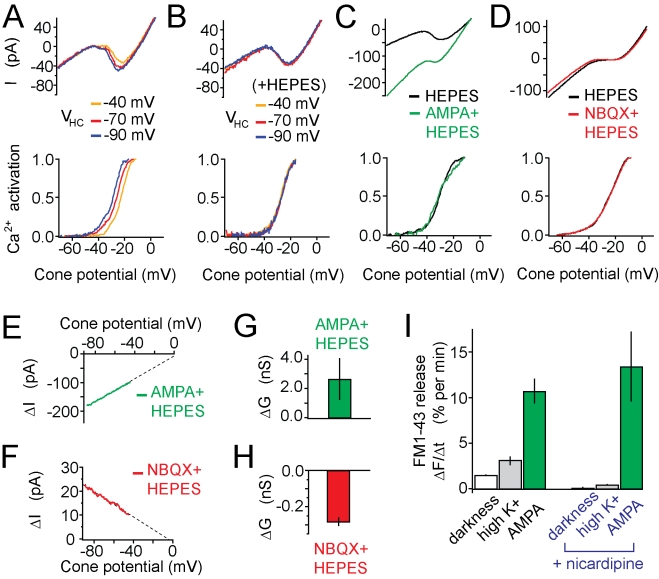
Ionic currents in cones modulated by negative and positive feedback. (A) Changing the voltage of an HC (V_HC_) shifts the activation of the voltage-gated Ca^2+^ current (I_Ca_) in a cone, indicative of negative feedback. Data for (A) and (B) were from simultaneous patch clamp recordings from a synaptically connected HC and a cone in a slice from salamander retina. Top: Current-voltage (I-V) curves at three different values of V_HC_. Bottom: I_Ca_ activation curves derived from these I-V curves (see Methods). I-V curves were generated by ramp depolarizations from −90 to 0 mV (0.5 mV/ms). I_Ca_ activation curves were obtained by subtracting the linear leak current from the total membrane current during the ramp. The resulting leak-subtracted I_Ca_ was normalized to the maximal I_Ca_ and plotted only over the voltage range where channels are activating (e.g., from −70 to −20 mV). Data are reported as mean ± SEM. (B) Same as (A), except with HEPES (10 mM) added to bath solution to block negative feedback. (C) AMPA (20 µM) elicits a voltage-independent conductance that increases with hyperpolarization. HEPES (10 mM) was added to the bath solution to block negative feedback. Traces represent average currents from five cones. Data for panels (C–H) were from patch clamp recordings from cones in flat-mounted salamander retinas. (D) NBQX (10 µM) reduces the voltage-independent conductance. HEPES was again added to the bath solution to block negative feedback. Traces represent average currents from 13 cones. (E, F) I-V curve of the voltage-independent conductance modulated by AMPA or NBQX. Difference currents were calculated from data in panels (C) and (D). Dashed lines are extrapolated linear fits to show reversal potentials. (G, H) Quantification of the average effect of AMPA (*n* = 5) or NBQX (*n* = 13) on the voltage-independent conductance. (I) L-type Ca^2+^ channels are not required for positive feedback. Left: The FM1-43 release rate in darkness is accelerated by adding high K^+^ (*n* = 9) or 20 µM AMPA (*n* = 16). Addition of nicardipine (100 µM) suppressed release in darkness (*n* = 7) and in high K^+^ (*n* = 3) but not in AMPA (*n* = 4).

We used a ramp depolarization in cones to activate voltage-gated Ca^2+^ channels, which generated an inward current at potentials more positive than −40 mV (Figure 6A, top). We computed the activation curve of the Ca^2+^ channels (Figure 6A, bottom). Hyperpolarizing the HC increases the cone Ca^2+^ current and shifts the activation curve to more negative potentials, whereas depolarizing decreases the current and shifts the curve to more positive potentials. Substituting extracellular HCO_3_ with HEPES prevents the HC-induced shift in activation (Figure 6B), as shown previously [11],[17]. Hence, HEPES blocks the electrophysiological consequence of negative feedback from HCs to cones.

To selectively examine the consequence of positive feedback, we added HEPES to the extracellular saline and recorded from cones in flat-mounted retinas instead of slices. Bath application of AMPA elicited an inward current that increased with hyperpolarization (Figure 6C) even below the activation range of voltage-gated Ca^2+^ channels. In fact, AMPA had no effect on the activation curve of the Ca^2+^ channels. Instead, AMPA elicited a current that changed linearly with voltage.

While AMPA elicited an *inward* current, the AMPA-receptor antagonist NBQX elicited a small, but significant *outward* current, also evident below the activation range of the voltage-gated Ca^2+^ channels (Figure 6D). The current versus voltage relationships of the AMPA- and the NBQX-elicited currents were linear below −40 mV (Figure 6E,F), consistent with regulation of voltage-insensitive channels. The extrapolated reversal potential of the AMPA and NBQX responses were −0.6±10 mV and −4.8±12 mV, respectively, with AMPA causing an increase in membrane conductance (Figure 6G) and NBQX causing a small but significant decrease (*p*<0.05) (Figure 6H). The nature of the channels that underlie this conductance is unknown, but the observation that AMPA leads to rise in Ca^2+^ in the cone terminal (Figure 5) is suggestive of a non-selective cation channel that is permeable to Ca^2+^. However, we cannot exclude the possibility that the voltage-independent channels are Ca^2+^-activated rather than Ca^2+^-permeable.

In summary, positive feedback leads to activation of a voltage-independent conductance in cones, distinct from negative feedback, which modulates a voltage-dependent Ca^2+^ conductance. Supporting this conclusion, we found that the nicardipine, a blocker of cone voltage-gated Ca^2+^ channels [39], had no effect on AMPA-induced FM1-43 release from anole cones but did block release in darkness and after exposure to high K^+^ saline (Figure 6I), both of which depolarize cones. The two forms of feedback can also be distinguished using HEPES, which blocks negative feedback but not positive feedback.

### Raising Ca^2+^ in HCs Triggers Positive Feedback

Our results thus far suggest that the effect of AMPA on cone release is mediated by HCs. However, the results of Figure 6 suggest that manipulation of HC voltage cannot completely recapitulate the effects of iGluR agonists and antagonists on cone release. Assuming that HCs are the source of positive feedback, something other than voltage must trigger retrograde signaling to photoreceptors.

Ca^2+^ seems a likely candidate. There is evidence that HCs contain Ca^2+^-permeable glutamate receptors [40] and glutamate application has been shown to elicit a rise in internal Ca^2+^ in HCs that does not involve influx through voltage-gated Ca^2+^ channels or release from internal stores [41],[42]. Moreover, glutamate receptors on HC dendrites are located adjacent to the cone terminals [43], ideally positioned to trigger a Ca^2+^-dependent feedback signal. To test this hypothesis, we applied philanthotoxin-74 (PhTx), a blocker of Ca^2+^-permeable AMPA receptors [44]. As expected, PhTx significantly decreased FM1-43 release from dark-adapted cones by 49%±17% (*p*<0.05, Figure S4A). Moreover, PhTx blocked AMPA-accelerated release from cone terminals by 68%±17% (*p*<0.01, Figure S4B), supporting the involvement of Ca^2+^-permeable AMPA receptors in positive feedback.

To confirm that glutamate can increase Ca^2+^ in HCs, we carried out current-clamp recordings from cells in slices of salamander retina and introduced through a patch pipette the Ca^2+^ indicator dye Ca^2+^-Orange (Figure S5A). Local extracellular two-photon uncaging of MNI-glutamate [37] led to an increase in Ca^2+^ in the portion of the dendritic tree immediately adjacent to the uncaging area (within 2 µm), but not in more distant dendrites (5B,C). This tight localization of the Ca^2+^ signal suggests that synaptic release of glutamate from photoreceptors would also result in a spatially localized rise in Ca^2+^ within an HC.

**Figure 7:**
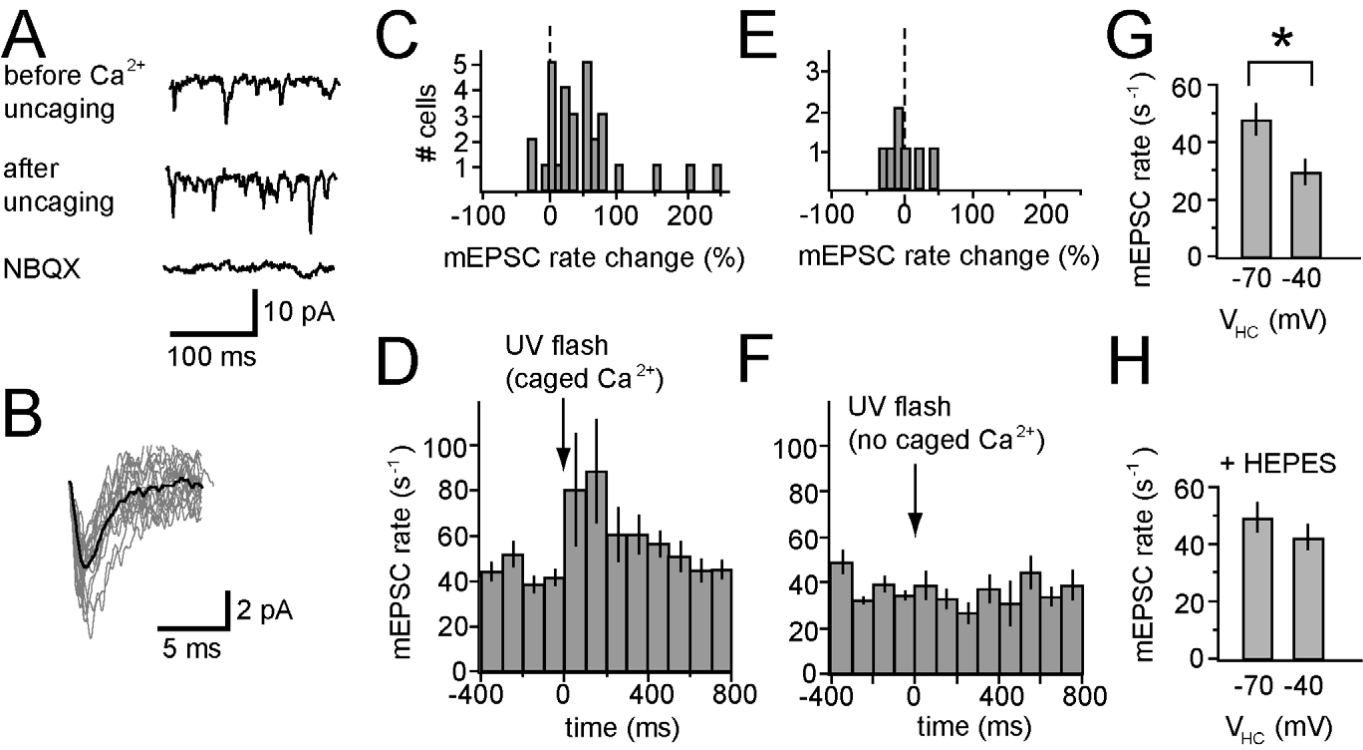
Uncaging Ca^2+^ in HCs increases glutamate release from photoreceptors. (A) Spontaneous inward currents recorded from an HC before (top trace) and after (middle trace) UV flash photolysis of caged Ca^2+^ (DM-nitrophen). Uncaging Ca^2+^ appeared to increase the frequency of mEPSCs. Spontaneous inward currents were eliminated after blocking AMPA receptors with 10 µM NBQX (bottom trace), identifying them as glutamatergic mEPSCs. (B) Average mEPSC waveform used for template matching (dark trace) and 10 individual events that were classified as mEPSCs. (C) Histogram showing distribution of HCs exhibiting increases or decreases in peak mEPSC rate after Ca^2+^ uncaging. (D) Time course of the change in mEPSC rate after Ca^2+^ uncaging. The time course reflects the average mEPSC rate from 31 HCs. (E) Histogram showing distribution of HCs exhibiting increases or decreases in peak mEPSC rate after UV flash when DM-nitrophen was absent from the pipette solution. (F) Time course mEPSC rate after UV flash, in the absence of DM-nitrophen. The time course reflects the average mEPSC rate from seven HCs. (G) Depolarization of HCs from −70 mV to −40 mV results in a statistically significant (*p*<0.05) decrease in average mEPSC rate (*n* = 4). (H) Depolarization of HCs from −70 mV to −40 mV fails to cause a significant increase in mEPSC rate after addition of HEPES (10 mM) to the bathing medium (*n* = 4).

To determine whether a rise in Ca^2+^ can trigger retrograde signaling from HCs, we elevated intracellular Ca^2+^ in an HC and asked whether this could increase neurotransmitter release from photoreceptors. We introduced caged Ca^2+^ (DM-nitrophen) via a patch pipette into an HC in a salamander retinal slice and used UV light to trigger photolysis and elevate internal Ca^2+^. A brief (1 ms) uncaging light flash triggered an increase in the frequency of small spontaneous inward currents in HCs (Figure 7A). The inward currents were blocked by NBQX, identifying the events as glutamatergic miniature excitatory postsynaptic currents (mEPSCs), which have been shown previously to result from photoreceptor vesicular release [45]. Salamander HCs receive input from rods and cones, so mEPSCSs could be generated by either cell type.

We quantified the rate of these events before and after Ca^2+^ uncaging in 31 HCs. To identify mEPSCs we used a template matching procedure that compared the waveform of individual events to the average mEPSC (Figure 7B). After Ca^2+^ uncaging, the peak mEPSC rate increased in 74% of cells, decreased in 10% of cells, and did not change in 16% of cells (Figure 7C). Overall, the average mEPSC rate increased 86%±29% in the 200 ms following uncaging, which was statistically significant (*p*<0.05). In HCs without DM-nitrophen, the flash caused no significant change in the mEPSC rate (Figure 7E,F). The increase in mEPSC rate was rapid, appearing within 100 ms after the uncaging flash (Figure 7C), and persisted for several hundred milliseconds, consistent with the expected decay of the cytoplasmic Ca^2+^ transient [46]. These results suggest that Ca^2+^ is sufficient for triggering the retrograde signal that accelerates vesicular glutamate release from photoreceptors. In addition, because Ca^2+^ was uncaged within an individual HC, these results identify HCs as a source of positive feedback.

Depolarization of HCs decreased the mEPSC rate, opposite to the effect of Ca^2+^ uncaging. After depolarizing from −70 to −40 mV the mEPSC rate declined by 38%±17% (*p*<0.05) (Figure 7G). This voltage span approximates the physiological operating range of the HC in light versus darkness. The decline in mEPSC rate with HC depolarization is consistent with negative feedback, which decreases neurotransmitter release from photoreceptors. HEPES inhibits negative feedback [11],[12],[35] and after adding HEPES, HC depolarization decreased the mEPSC rate by only 8%±12%, no longer statistically significant (Figure 7H). Taken together, these findings indicate that depolarization of the HC leads to a decrease in neurotransmitter release from photoreceptors, whereas raising Ca^2+^ leads to an increase in neurotransmitter release.

We also attempted to detect positive feedback by recording membrane current in an individual cone while uncaging Ca^2+^ in an individual HC. Positive feedback should elicit a voltage-independent conductance in the cone, as was elicited by AMPA applied on the entire flat-mounted retina (see Figure 6). However, Ca^2+^ uncaging had no significant effect (unpublished data). Each salamander cone is contacted by ∼12 HCs [47], so the magnitude of the feedback effect on a single cone from manipulating a single HC should only be 1/12 of the total feedback effect, perhaps too small to detect.

## Discussion

### HCs Transmit a Positive Feedback Signal to Photoreceptors

The findings presented in this article reveal a previously unknown positive feedback synapse onto cone photoreceptors. Our results indicate that HCs are the source of this positive feedback signal. HCs possess the type of glutamate receptors that we implicate in positive feedback (AMPA receptors) and these receptors are located on HC dendrites that invaginate the cone terminal adjacent to sites of synaptic vesicle exocytosis. Selective laser ablation of cells in the HC layer of the INL eliminates positive feedback. Retinal slice experiments suggest that cells that project laterally for >200 µm are required, consistent with the cytoarchitecture of HCs. Finally, our mEPSC analysis indicates that neurotransmitter release from photoreceptors can be evoked by uncaging Ca^2+^ within an individual HC, identifying HCs as the source of positive feedback. This shows that a rise in Ca^2+^ in an HC is *sufficient* for triggering positive feedback to photoreceptors, but we do not yet have the tools to confirm that a rise in Ca^2+^ in HCs is *necessary* for positive feedback.

Several special features of the synaptic connection between HCs and cones may help explain why positive feedback has evaded notice over the past four decades. First, positive feedback onto a cone cannot be evoked simply by depolarizing a synaptically connected HC, the standard test for synaptic connectivity. Second, positive feedback is compromised by making transverse slices of the retina, a procedure that is a near-necessity for making electrophysiological recordings between HCs and other neurons. Third, without a means for selectively eliminating positive feedback, its effects could easily be misattributed to a higher intrinsic gain of the synaptic release machinery in cones.

The discovery of positive feedback helps explain a long-standing puzzle about synaptic signaling in the outer retina. Kainate and other selective iGluR agonists hyperpolarize On-bipolar cells in the intact retina [48],[49] but not in retinal slices [50]–[52]. On-bipolar cells in slices continue to exhibit a robust response to glutamate, but this can be completely attributed to mGluRs [53], which are unaffected by kainate. Our results may help explain the indirect action of iGluR agonists: They trigger HC-mediated positive feedback onto rods and cones, increasing their release of glutamate, which leads to hyperpolarization of the On-bipolar cell. An iGluR-elicited signal in amacrine cells may be communicated to On-bipolar cells through GABA receptors, also contributing to the hyperpolarization [51].

Our results suggest that positive feedback applies not only to cones but also to rods. Most of our optical studies utilized the all-cone retina from anoles, but in retinas containing both rods and cones (including zebrafish, salamander, and rabbit) we noticed no difference in the AMPA-elicited acceleration of neurotransmitter release in rods and cone terminals interspersed in the OPL. Rods and cones are electrically coupled through gap junctions [54], so it is possible that AMPA-elicited enhancement of release from rods is an indirect consequence of signals originating in cones. It is also possible that glutamate released at photoreceptor synapses “spills over” to affect other photoreceptor synapses, contributing to enhanced release from both rods and cones. However, AMPA accelerates release from rod photoreceptors in the gecko retina, which has no true cones. HC-mediated *negative* feedback has recently been demonstrated to occur in rods as well as cones [5], so it seems likely that the positive feedback signal is also communicated to both photoreceptors.

### Searching for the Mechanism of Positive Feedback

**Figure 8:**
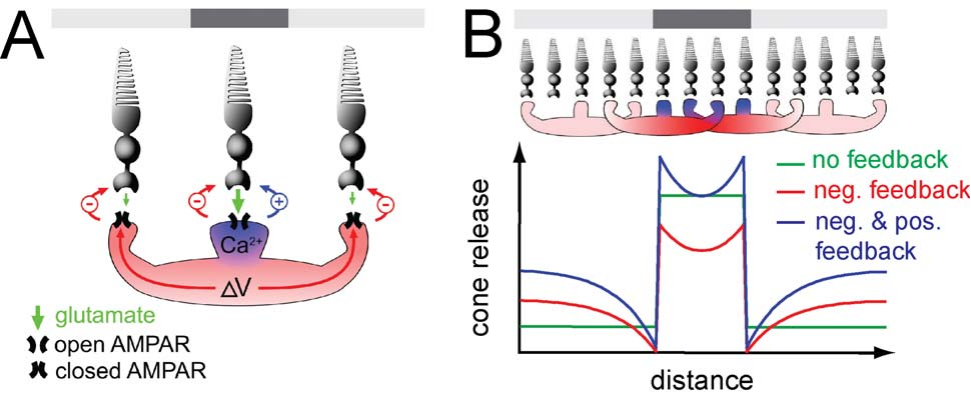
A conceptual model of positive feedback in the outer retina. (A) Diagram depicting the differential spread of positive and negative feedback within an HC. The top bar denotes the illumination pattern. A cone depolarized in darkness will release glutamate, activating AMPA receptors (APMARs), causing depolarization and Ca^2+^ influx. The rise in Ca^2+^ is restricted to the specific dendrite that contacts the cone, and the resulting positive feedback is localized to that cone. The depolarization spreads electrotonically through the HC, resulting in negative feedback from all of the dendrites. (B) Model simulations of the effect of feedback on synaptic release from a linear array of cones exposed to a dark spot on a non-saturating light background (see Methods). The positive feedback signal (blue) is localized to HC dendrites in contact with dark cones while the negative feedback signal (red) electrotonically spreads through the HCs. Traces show simulated cone release with no feedback (green), with negative feedback, (red), and with equally weighted negative and positive feedback (blue).

Figure 8A shows our proposed outline of the positive feedback process. First, glutamate released from a cone activates Ca^2+^-permeable AMPA receptors on an HC, leading to Ca^2+^ influx. This results in an increase in cytoplamsic Ca^2+^. Next, we propose that intracellular Ca^2+^ in the HC triggers the release of a retrograde messenger that acts on the cone terminal. Finally, this messenger causes an increase in cone terminal Ca^2+^ that accelerates neurotransmitter release.

The two most important unanswered questions are: (1) What is the identity of the retrograde messenger? (2) What is the receptor for that messenger that leads to a rise of intracellular Ca^2+^ in cones? Our experiments rule out many conventional neurotransmitters being the retrograde messenger. Positive feedback persists after adding GABA, glycine, or dopamine to the retina, ruling out these neurotransmitters. In fact, EM studies show that HC dendrites lack accumulations of synaptic vesicles and plasma membrane specializations found at active zones [38], making it unlikely that a conventionally secreted neurotransmitter is involved.

However, neurotransmitters can be released by means other than synaptic vesicle exocytosis. Activation of plasma membrane transporters on HCs can lead to the efflux of GABA [55] and protons [56],[57], but our experiments rule out either of these being the positive feedback signal. Nitric oxide (NO) diffuses across biological membranes and serves as a retrograde synaptic messenger in the brain [58]. HCs possess NO synthase (NOS) [59],[60], cones possess the NO effector enzyme soluble guanylate cyclase (sGC) [61],[62], cone terminals possess cyclic nucleotide-gated (CNG) channels, and Ca^2+^ influx through these channels can support release [63]. Moreover, NO donors increase exocytosis from isolated cones [64] and NOS or sGC inhibitors decrease release [63]. These observations might seem to make NO a likely candidate. However, we found that positive feedback persists after application of an NO donor, inhibitors of NOS, or inhibitors of sGC (Figure S6A). The ineffectiveness of these reagents argues against NO as the positive feedback transmitter.

Phospholipid-derived molecules are another class of potential retrograde messengers. Arachidonic acid and other polyunsaturated fatty acids are released by the retina in response to light [65],[66], however these compounds inhibit voltage-gated Ca^2+^ channels in photoreceptors [67], different from the actions of the positive feedback transmitter. Endocannabinoids, including anandamide and 2-arachidonoyl glycerol (2-AG), are also found in the retina and there is evidence that they modulate voltage-gated conductances in cones [68]. We find that anandamide does not activate a voltage-independent conductance in cones (Figure S6C), suggesting that it is not the positive feedback transmitter. There are many other phospholipid-derived molecules that can serve as messengers between cells. For now, the transmitter that mediates positive feedback from HCs to cones is unknown, but hopefully, this mystery will be solved more quickly than the mechanism of negative feedback.

The retrograde messenger activates a voltage-independent conductance and increases intracellular Ca^2+^ in cones, but we do not yet know the channel(s) that are responsible for these events. Several types of Ca^2+^-permeable non-selective cation channels are found in photoreceptor terminals, including CNG channels [63],[64] and TRPC channels [69]. The voltage-independent channel may be the conduit for Ca^2+^ entry, but we cannot exclude the possibility that activation of these channels is an indirect consequence of Ca^2+^ elevation through some other type of channel rather than being the cause of Ca^2+^ elevation. Our results exclude several other possible routes of Ca^2+^ entry. Voltage-gated Ca^2+^ channels can be excluded because AMPA accelerates release in nicardipine (Figure 6I). Ca^2+^ release from internal stores seems unlikely because reagents that interfere with this process do not inhibit AMPA-accelerated release (Figure S6B).

### Functional Consequences of Positive and Negative Feedback

The cone synapse encodes information about light intensity by modulating its rate of vesicular neurotransmitter release. Because vesicular release is quantal, the encoding capacity of the cone synapse is limited by the maximal release rate (i.e., in darkness) [14]. Any process that decreases the maximal release rate will degrade the representation of light intensity by the cone synapse, which in turn will degrade the performance of the visual system as a whole.

Negative feedback from HCs enhances the neural representation of spatial contrast as an array of photoreceptors transmits a visual image to bipolar cells. However, the benefit of contrast enhancement comes at a cost: negative feedback lowers the maximal release rate and therefore reduces the dynamic range of the cone synapse. This compresses the neural representation of a visual image, counteracting enhanced contrast sensitivity. By boosting neurotransmitter release from cones, positive feedback may recoup the dynamic range lost to negative feedback. The factors that prevent positive feedback from causing runaway excitation remain to be determined.

Our results indicate that positive feedback temporally overlaps with negative feedback and might play a role in the neural representation of visual stimuli. While the precise time course of positive feedback remains to be determined, the rise in intracellular Ca^2+^ in an HC peaks within 1 s of glutamate application (Figure S5C), and the acceleration of photoreceptor transmitter release peaks within 100 ms of HC Ca^2+^ elevation (Figure 7D). These values are over-estimates that are limited by the temporal resolution of our recording methods. In comparison, negative feedback peaks within 100 ms of the onset of light [3] and is maintained indefinitely with persistent illumination.

We propose that the signals that give rise to positive and negative feedback spread differently through an HC, which may explain why the signals do not simply cancel out (Figure 8B). Our results suggest that positive feedback acts locally, occurring only where HC dendrites receive direct excitatory input (Figure 5F,G). Immunocytochemistry shows that HCs express the calcium-binding proteins parvalbumin and CaBP-28K [70], and these proteins may limit the spread of Ca^2+^ to individual HC dendrites, making Ca^2+^-dependent positive feedback local. In contrast, negative feedback is controlled by the HC voltage, not by Ca^2+^. The voltage signal generated by synaptic current into individual dendrites spreads electrotonically, not only within a single HC but through the syncytium of HCs, distributing negative feedback over a large area [71],[72]. Thus our working hypothesis is that positive feedback is more spatially constrained than negative feedback.

It is perhaps counterintuitive, but if the two feedback signals were to spread differently, positive feedback would amplify, rather than suppress, contrast enhancement mediated by negative feedback. We explored the interplay between these two processes by constructing a model (see Methods) of how feedback might impact release from a linear array of cone terminals (Figure 8B). When a dark spot on a brighter background is projected on the retina, cones within the center of the spot are depolarized, maintaining their rate of glutamate release, while those in the surround are hyperpolarized, decreasing release. In the absence of negative feedback, the spatial profile of release from an array of cones mirrors the profile of the stimulus. Adding negative feedback enhances the representation of contrast by opposing the intrinsic response of cones. Release from terminals in the dark spot is decreased by depolarized HCs, while release from terminals in the brighter surround is increased by hyperpolarized HCs. Cone terminals at the light-dark edge receive inputs from HCs straddling the border. Because negative feedback spreads through HCs, cones near the edge will receive signals from both depolarized and hyperpolarized HCs and their effects will cancel, unleashing the full ability of light and dark to modulate cone release. If positive feedback were spatially constrained, it would scale release in direct proportion to the local synaptic output from individual cones. As a result, positive feedback could amplify cone release without sacrificing the contrast enhancement provided by negative feedback.

## Materials and Methods

### Dye Loading

All procedures were approved by the UC Berkeley Animal Care and Use Committee. Retina were isolated as described previously [14] from lizards (*Anolis segrei*), tiger salamanders (*Abystoma tigrinum*), tokay geckos (*Gecko gecko*), zebrafish (*Danio rerio*), and New Zealand white rabbits maintained on a 12∶12 light:dark cycle. Retinas were isolated at 21°C in complete darkness with the aid of an IR converter. Isolated retinas were bathed in saline containing (in mM) for lizard: NaCl 149, KCl 4, CaCl_2_ 1.5, MgCl_2_ 1.5, HEPES 10, Glucose 10, pH 7.4; for salamander: NaCl 110, KCl 2, CaCl_2_ 2, MgCl_2_ 1, HEPES 10, Glucose 10, pH 7.4; for gecko: NaCl 160, KCl 3.3, CaCl_2_ 1.5, MgCl_2_ 1.5, HEPES 10, Glucose 10, pH 7.4. For experiments on lizard retinas involving bicarbonate buffered solution, isolation, and loading took place exclusively in bicarbonate buffered solution, containing (in mM): NaCl 124, KCl 4, CaCl_2_ 1.5, MgCl_2_ 1.5, NaHCO_3_ 20, Glucose 10, pH 7.5, adjusted after bubbling with 95% O_2_–5% CO_2_. For rabbit retinas, the saline solution contained: 1.9 g/L NaHCO_3_, 0.05 g/L kanamyacin sulfate, 8.8 g/L Ames powder (Sigma), bubbled with 95% O_2_–5% CO_2_.

For FM1-43 loading, retinas were mounted onto nitrocellulose filter paper RPE-side down, and bathed in saline containing 30 µM FM1-43 for 1–3 h followed by a 5 min wash with 1 mM Advasep-7, as described previously [13]. Retinal slices were prepared using a Stoelting tissue chopper. Retinas were mounted for imaging in a gravity fed perfusion chamber with a bath volume of ∼0.5 ml (Warner Instruments) and perfused with solution at 1 ml/min. Drugs were bath applied by perfusion. High K^+^ saline contained 50 mM KCl, iso-osmotically replacing NaCl.

For Ca^2+^ indicator dye loading, retinas were bathed for 2 h in saline containing 100 µM of either Oregon Green BAPTA-1 AM (OGB-1 AM) or, in some experiments, X-Rhod-1 AM (Molecular Probes). The loading solution contained 1% DMSO and 0.2% pluronic acid to enhance dye solubility and cell permeation, and retinas were gently agitated to encourage dye-permeation into the tissue. Dye-loaded retinas were mounted onto nitrocellulose filter paper RPE-side down after loading.

### Imaging

Retinas were imaged with a Zeiss 510 upright confocal microscope equipped with a MaiTai (Spectra Physics) mode-locked Ti:sapphire laser and a 20× or 40× water-immersion objective. The excitation wavelength was tuned to 860 nm (FM1-43), 800 nm (OGB-1), or 880 nm (X-Rhod-1). Laser intensity was 20–40 mW, averaged power. Images were acquired with Zeiss LSM software, and ImageJ software (rsb.info.nih.gov/ij) was used to analyze the average fluorescence of the OPL.

FM1-43 fluorescence in the OPL of retinal flat-mounts was measured by taking 5–6 images in a Z-stack spaced at 2 µm so that the OPL fell entirely within the Z-stack. Anole cone terminals are ∼6 µm in diameter, so in each stack the brightest focal plane could be identified as containing the center of the cone terminals, and average intensity was measured from this focal plane. This allowed us to readjust our measurement at each time point to compensate for movement of the sample. When the retina was not perfectly flat, the image was divided into quadrants and the measurements were made from the brightest image for each quadrant. The pixel dimension was 0.82×0.82 µm (256×256 pixels) and the dwell time per pixel was 3.2 µs (493 ms per image).

Fluorescence was normalized to 1.0 at the start of experiments. Dye release rates were calculated from the fluorescence decrements between images in time series. Statistical comparisons were performed using unpaired two-tailed Student's *t* tests in Excel (Microsoft). In previous studies we background subtracted OPL measurements with respect to the fluorescence of the INL, which exhibits no light-dependent change in fluorescence over the recording period of ∼20 min [14]. However, in this study we could not be certain that glutamate receptor agonists have no effect on FM1-43 fluorescence in the INL, so no background subtraction was used. Consequently, the rate of the normalized FM1-43 fluorescence decline is slower here than in previous reports [14],[73]. Graphing and curve fitting was performed using Igor Pro (Wavemetrics). Unless noted otherwise, variability among data throughout the article is represented as mean ± SEM.

### Ablation and Agonist-Uncaging

To ablate cell layers in retinal slices, the Ti:sapphire laser power was increased to maximal (∼2 W average output) and the laser was scanned in a line 400–500 µm across the slice surface at the layer of the retina chosen for ablation, at a speed of 500 µm/ms. The line scan was repeated 8 times, before the focus was moved in the z-direction. This was repeated at depth intervals of 2 µm, to a final depth of 30 µm into the slice.

For experiments involving NVOC-AMPA uncaging, light from a mercury lamp was focused through the microscope objective onto the plane of the OPL. Light was filtered through a Zeiss FS02 filter set (EM_max_ = 365 nm) and controlled by electronic shutter. To uncage AMPA within a small spot, the microscope field stop was closed, producing a spot of UV light with a Gaussian profile and a FWHM diameter of 100 µm, determined by photobleaching of FM1-43 absorbed onto filter paper.

For MNI-glutamate uncaging, the mode-locked Ti:sapphire laser was tuned to 720 nm light (∼50 mW). After adding 2 mM MNI-glu to the bath solution, the laser was scanned over a 2×2 µm section of the HC dendrite for <100 µs. Calcium Orange dye was excited with a 543 nm HeNe laser, and images were acquired at 1 s intervals using a Zeiss 510 upright confocal microscope.

### Electrophysiology

Retinal slices from the larval tiger salamander *Ambystoma tigrinum* were prepared as previously described [22] using procedures approved by the UNMC Institutional Animal Care and Use Committee. The standard bath solution contained (in mM): 101 NaCl, 22 NaHCO_3_, 2.5 KCl, 0.5 MgCl_2_, 2 CaCl_2_, 9 glucose. The pH was ∼7.35 after bubbling with 95% O_2_/5% CO_2_. Where indicated, pH buffering capacity was increased by adding 10 mM HEPES. Whole-cell voltage-clamp recordings were obtained from cones or horizontal cells using 10–15 MΩ patch pipettes pulled from borosilicate glass. The standard pipette solution contained (in mM): 48 CsGluconate, 42 CsCl, 9.4 TEACl, 1.9 MgCl_2_, 9.4 MgATP, 0.5 GTP, 5 EGTA, 32.9 HEPES (pH 7.2). For MNI-glutamate uncaging on horizontal cells, the pipette solution contained 0.5 EGTA and 100 µM Calcium Orange. For caged Ca^2+^ experiments with horizontal cells, the pipette solution consisted of (in mM): 40 CsGluconate, 20 CsGlutamate, 40 CsHEPES, 10 TEACl, 10 DM-nitrophen, 8 CaCl2, 1 MgCl_2_, 2 DPTA, 5 NaATP, 0.5 mM GTP (pH 7.2). Resting [Ca^2+^] prior to photolysis was <200 nM as determined from aliquots using Fura-2. DM-nitrophen was photolyzed by flashes of UV light derived from a Xenon arc flash lamp (Rapp Optoelectronic). Cones were voltage clamped at −70 mV and horizontal cells at −60 mV using a Multiclamp or Axopatch 200B patch-clamp amplifier. Data were acquired with a Digidata 1322 interface and pClamp 9.2 software (Axon Instruments). mEPSCs were analyzed using the template search Clampfit 10.2 (Molecular Devices). Statistical comparisons were performed using paired two-tailed Student's *t* tests in Excel.

### Modeling Feedback

An array of photoreceptors was stimulated with a dark spot on a brighter background. Photoreceptors were assigned arbitrary intrinsic release rates of 1 in darkness and 0.2 in light. To model negative feedback, release from many photoreceptors was integrated by each post-synaptic HC to produce an unscaled HC voltage that in turn regulated feedback, according to the function:
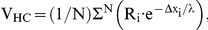
where N is the number of presynaptic cones, R_i_ the release rate of the ith cone, Δx_i_ the distance between the ith cone and the HC center, and λ the length constant of voltage spread in HCs. Release after feedback was calculated as R_neg_ =  R_i_ − C_neg_• (V_HC_−R_min_), and R_pos_ =  C_pos_•R_neg_, where C_neg_ and C_pos_ are arbitrary scaling constants determining the strength of feedback, which were both set to 1.5.

## Supporting Information

Figure S1 AMPA increases vesicular release from photoreceptors in disparate vertebrate species. (A–D) Fluorescence images of FM1-43-loaded cone terminals in the outer plexiform layer of a flat-mounted retina of (A) zebrafish (*Danio rerio*), (B) tiger salamander (*Ambystoma tigrinum*), (C) rabbit (*Oryctolagus cuniculus*), and (D) gecko (*Gecko gecko*). FM1-43 loading was more uniform than it appears in some of the figures. This was because the retina was not perfectly flat, so the OPL was not in focus everywhere within the field of view (scale bar = 100 µm). (E–H) In each species, 20 µM AMPA increased the FM1-43 release rate by >2-fold as compared to darkness. *N* = 4 for each species.
(TIF)

Figure S2 AMPA increases release from cones in both light-adapted and dark-adapted retina. Anole retinas loaded with FM1-43 were light-adapted for 20 min with bright white light from a halogen bulb (10^7^ photons/µm^2^/s) prior to the application of 20 µM AMPA. The light was briefly extinguished every 2 min in order to image the terminals. Light decreased the FM1-43 release rate significantly (*n* = 3) as compared to darkness (*n* = 27), but light did not stop AMPA from increasing release (*n* = 3 light adapted, *n* = 16 dark adapted), indicating that AMPA is dominant in increasing release when cones are hyperpolarized by light.
(TIF)

Figure S3 Neurotransmitters released by interplexiform cells (IPCs) do not mediate AMPA-accelerated release from cones. To ascertain whether IPCs might be the source of positive feedback onto cones, we asked whether AMPA could still accelerate the cone release rate after applying agonists or antagonists of dopamine or glycine receptors. AMPA acceleration of FM1-43 release from anole cone terminals was unaffected by dopamine (100 µM; *n* = 4) or glycine (1 mM; *n* = 2). The glycine receptor antagonist strychnine (1 µM; *n* = 2) did not significantly change AMPA-accelerated release (*p* = 0.25).
(TIF)

Figure S4 Positive feedback operates through Ca^2+^-permeable AMPA receptors. (A) Bath addition of 100 µM philanthotoxin-74 (PhTx), a blocker of Ca^2+^-permeable AMPA receptors (CP-AMPARs), significantly slows release from cone terminals in darkness (*n* = 5, *p*<0.05), suggesting that ambient glutamate boosts release by stimulating CP-AMPARs. (B) 20 µM AMPA markedly increases the release rate from cones (*n* = 16). 100 µM PhTx significantly reduces the effect of AMPA by 68%±17% (*n* = 5, *p*<0.01).
(TIF)

Figure S5 Local photolysis of caged glutamate results in local elevation of intracellular Ca^2+^ in an HC dendrite. (A) Fluorescent image of an HC in a salamander retinal slice. The cell was filled with the Ca^2+^ indicator dye Ca^2+^ Orange, and boxes represent regions of interest where fluorescence intensity was measured. MNI-glutamate was uncaged by 2-photon photolysis in the area denoted in Region 2. Scale bar = 10 µm. (B) Difference images showing that uncaging of glutamate elicits an increase in Ca^2+^ selectively in Region2, but not in Regions 1 or 3. Images of the three regions were equally contrast enhanced for the purposes of display. (C) Time course of the fluorescent changes in the three regions.
(TIF)

Figure S6 Evidence against NO or anandamide as positive feedback transmitters. (A) Drugs affecting the NO signaling pathways fail to suppress 20 µM AMPA-accelerated FM1-43 release from flat-mounted anole retina. Drugs indicated are the NO-donor NOR4 (100 µM; *n* = 3), the sGC inhibitor ODQ (100 µM; *n* = 3), and the NOS inhibitors L-NMMA (100 µM; *n* = 2) and 7-NI (100 µM; *n* = 3). (B) Drugs affecting Ca^2+^ mobilization from internal stores fail to suppress AMPA-accelerated FM1-43 release. Drugs indicated are ryanodine (100 µM; *n* = 3), dantrolene (50 µM; *n* = 2), and thapsigargin (2 µM; *n* = 3), xestospongin (1 µM; *n* = 3). (C) The endocannabinoid anandamide does not alter conductance in cones. I-V curve from a patch-clamped cone in a flat-mounted salamander retina. Anandamide (100 µM) did not affect the voltage-independent conductance, nor did it change the Ca^2+^-activation curve, suggesting that anandamide is not the mechanism of positive or negative feedback.
(TIF)

## Abbreviations

CBO: carbenoxolone
CNG: cyclic nucleotide-gated
HC: horizontal cell
INL: inner nuclear layer
IPC: interplexiform cell
IPL: inner plexiform layer
mEPSC: miniature excitatory postsynaptic current
NO: nitric oxide
NOS: NO synthase
sGC: soluble guanylate cyclase
