# Supplementary figures and images for: A Positive Feedback Synapse from Retinal Horizontal Cells to Cone Photoreceptors
Source: PLoS Biol. 2011 May 3;9(5):e1001057. doi: 10.1371/journal.pbio.1001057 (PMC3086870; doi:10.1371/journal.pbio.1001057)

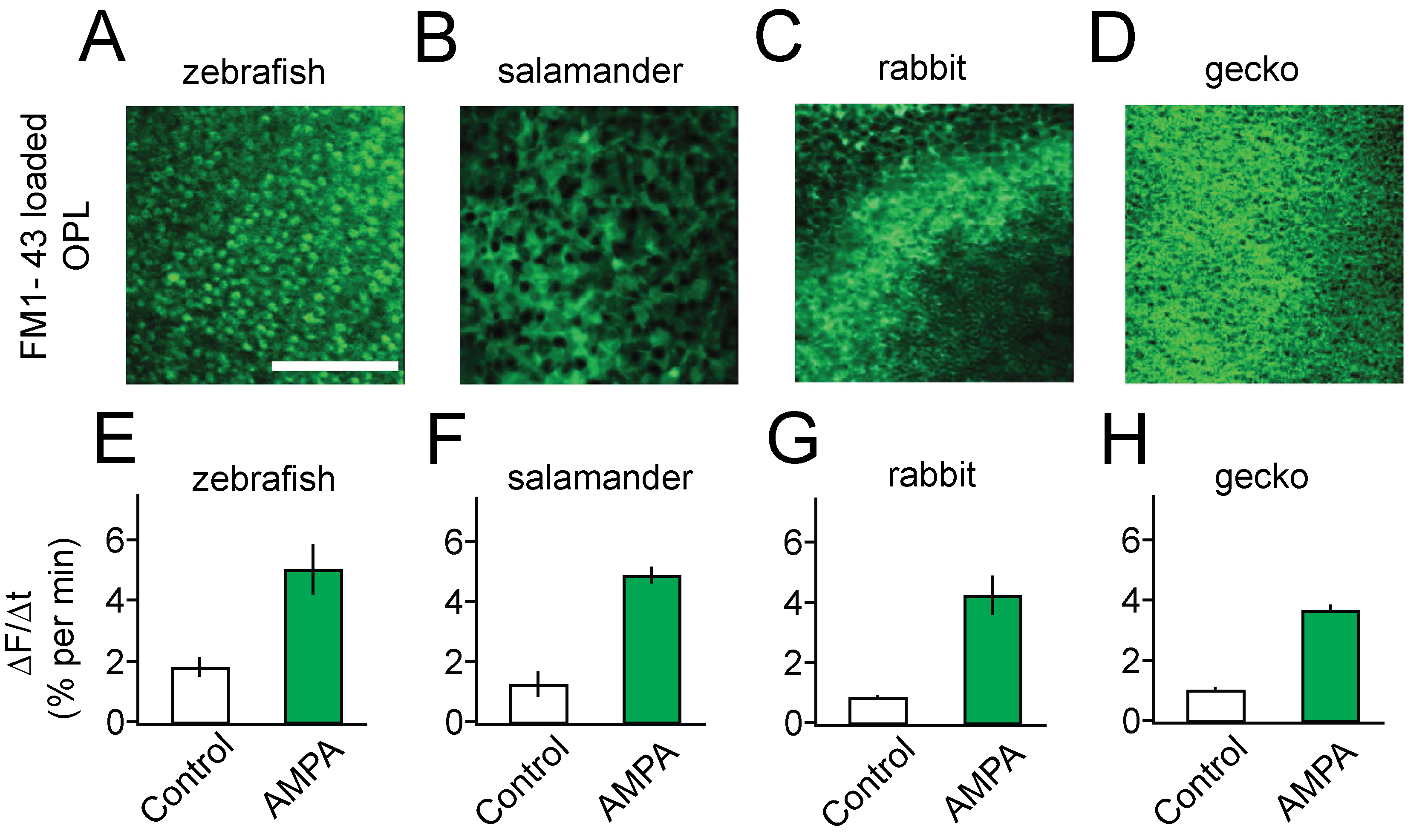

Supplement: Figure S1 — AMPA increases vesicular release from photoreceptors in disparate vertebrate species. (A–D) Fluorescence images of FM1-43-loaded cone terminals in the outer plexiform layer of a flat-mounted retina of (A) zebrafish (Danio rerio), (B) tiger salamander (Ambystoma tigrinum), (C) rabbit (Oryctolagus cuniculus), and (D) gecko (Gecko gecko). FM1-43 loading was more uniform than it appears in some of the figures. This was because the retina was not perfectly flat, so the OPL was not in focus everywhere within the field of view (scale bar = 100 µm). (E–H) In each species, 20 µM AMPA increased the FM1-43 release rate by >2-fold as compared to darkness. N = 4 for each species. (TIF) [file pbio.1001057.s001.tif]

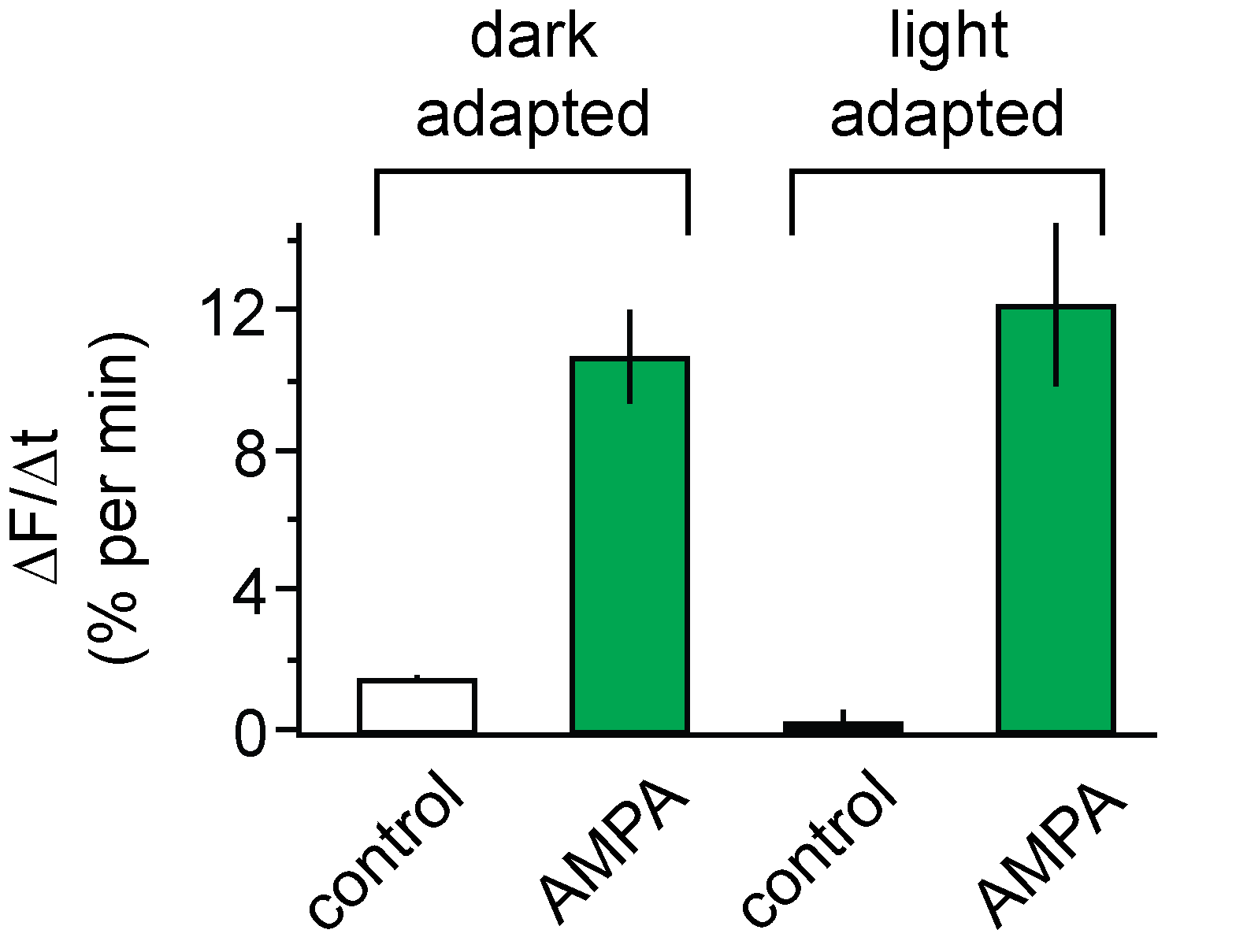

Supplement: Figure S2 — AMPA increases release from cones in both light-adapted and dark-adapted retina. Anole retinas loaded with FM1-43 were light-adapted for 20 min with bright white light from a halogen bulb (107 photons/µm2/s) prior to the application of 20 µM AMPA. The light was briefly extinguished every 2 min in order to image the terminals. Light decreased the FM1-43 release rate significantly (n = 3) as compared to darkness (n = 27), but light did not stop AMPA from increasing release (n = 3 light adapted, n = 16 dark adapted), indicating that AMPA is dominant in increasing release when cones are hyperpolarized by light. (TIF) [file pbio.1001057.s002.tif]

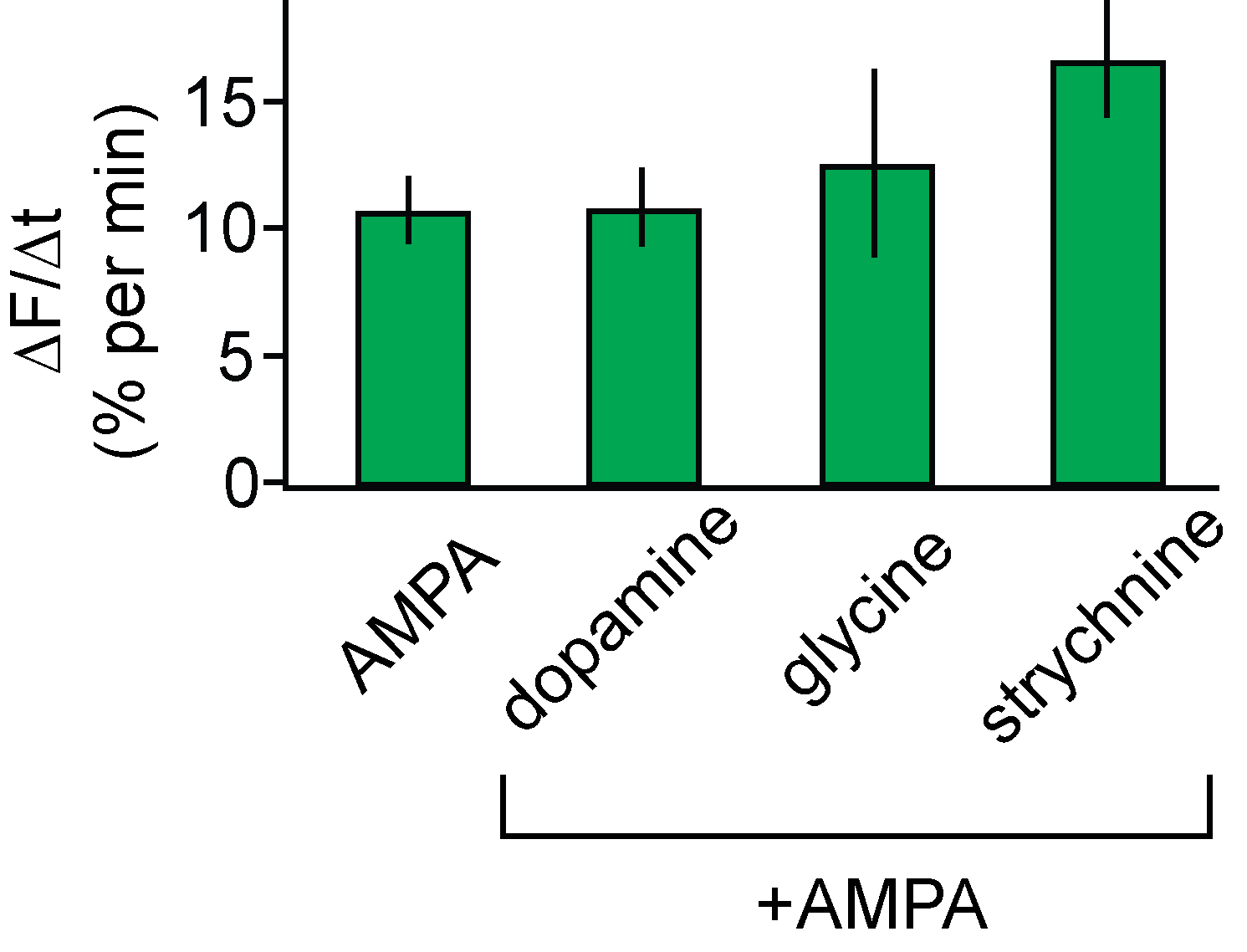

Supplement: Figure S3 — Neurotransmitters released by interplexiform cells (IPCs) do not mediate AMPA-accelerated release from cones. To ascertain whether IPCs might be the source of positive feedback onto cones, we asked whether AMPA could still accelerate the cone release rate after applying agonists or antagonists of dopamine or glycine receptors. AMPA acceleration of FM1-43 release from anole cone terminals was unaffected by dopamine (100 µM; n = 4) or glycine (1 mM; n = 2). The glycine receptor antagonist strychnine (1 µM; n = 2) did not significantly change AMPA-accelerated release (p = 0.25). (TIF) [file pbio.1001057.s003.tif]

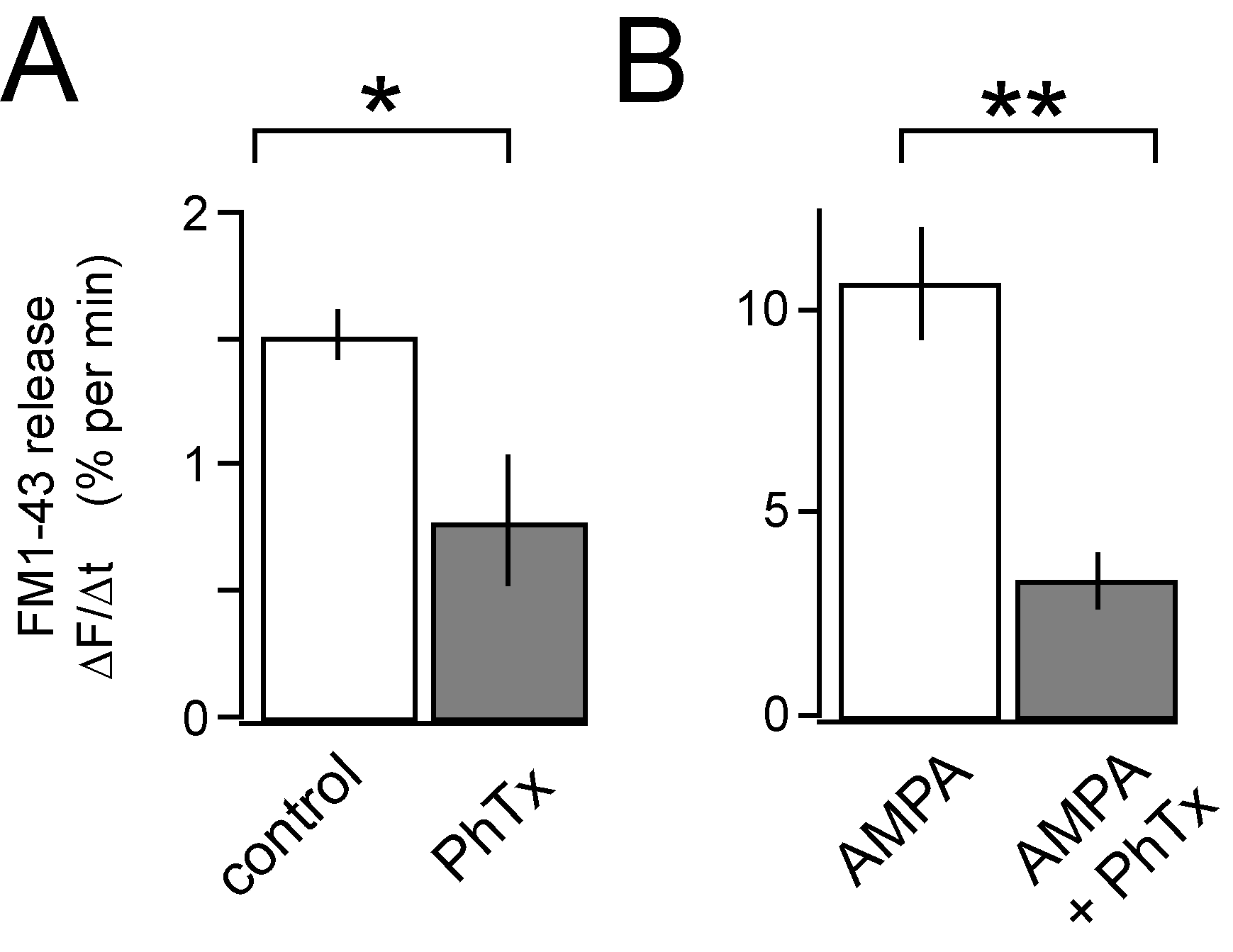

Supplement: Figure S4 — Positive feedback operates through Ca2+-permeable AMPA receptors. (A) Bath addition of 100 µM philanthotoxin-74 (PhTx), a blocker of Ca2+-permeable AMPA receptors (CP-AMPARs), significantly slows release from cone terminals in darkness (n = 5, p<0.05), suggesting that ambient glutamate boosts release by stimulating CP-AMPARs. (B) 20 µM AMPA markedly increases the release rate from cones (n = 16). 100 µM PhTx significantly reduces the effect of AMPA by 68%±17% (n = 5, p<0.01). (TIF) [file pbio.1001057.s004.tif]

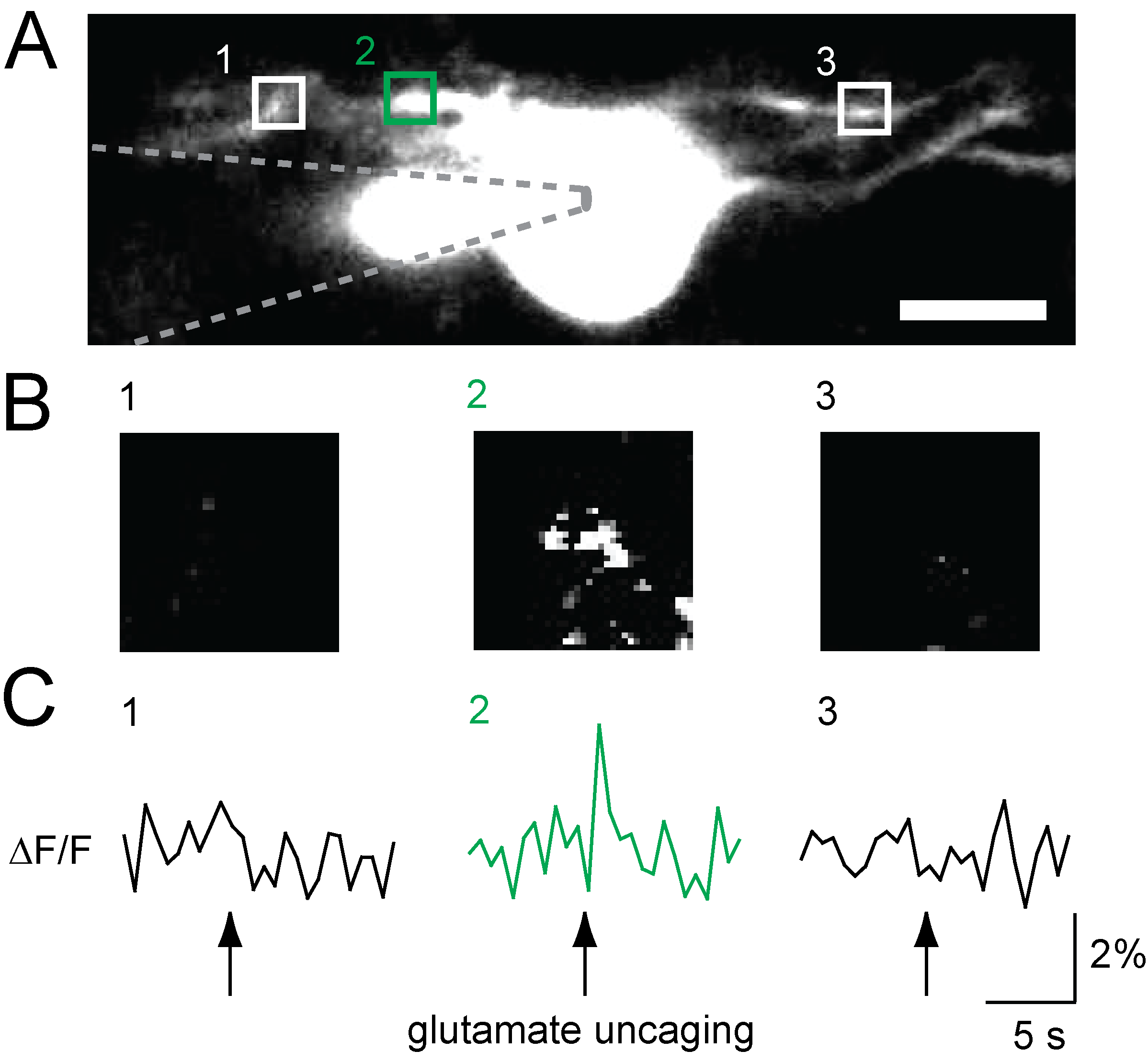

Supplement: Figure S5 — Local photolysis of caged glutamate results in local elevation of intracellular Ca2+ in an HC dendrite. (A) Fluorescent image of an HC in a salamander retinal slice. The cell was filled with the Ca2+ indicator dye Ca2+ Orange, and boxes represent regions of interest where fluorescence intensity was measured. MNI-glutamate was uncaged by 2-photon photolysis in the area denoted in Region 2. Scale bar = 10 µm. (B) Difference images showing that uncaging of glutamate elicits an increase in Ca2+ selectively in Region2, but not in Regions 1 or 3. Images of the three regions were equally contrast enhanced for the purposes of display. (C) Time course of the fluorescent changes in the three regions. (TIF) [file pbio.1001057.s005.tif]

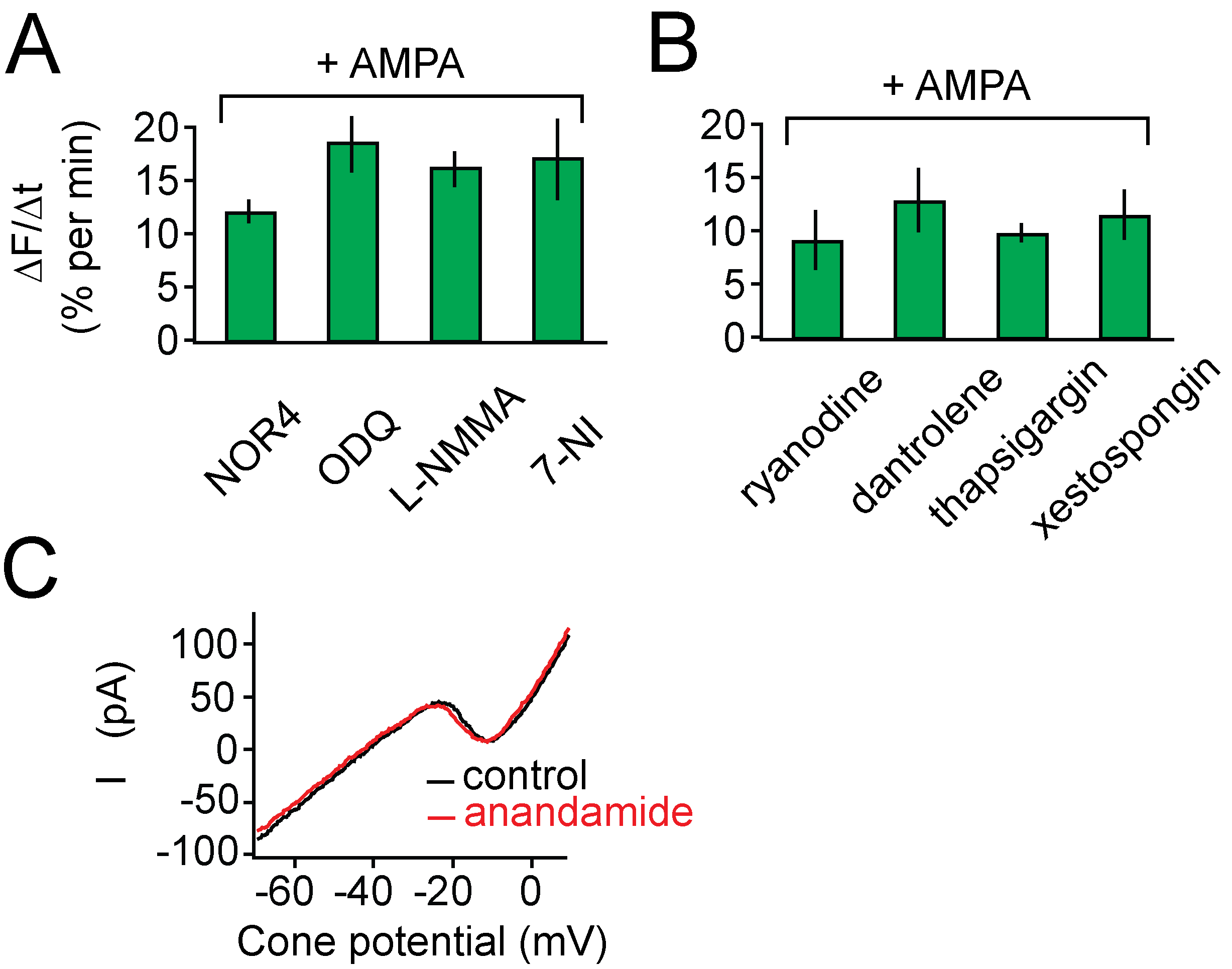

Supplement: Figure S6 — Evidence against NO or anandamide as positive feedback transmitters. (A) Drugs affecting the NO signaling pathways fail to suppress 20 µM AMPA-accelerated FM1-43 release from flat-mounted anole retina. Drugs indicated are the NO-donor NOR4 (100 µM; n = 3), the sGC inhibitor ODQ (100 µM; n = 3), and the NOS inhibitors L-NMMA (100 µM; n = 2) and 7-NI (100 µM; n = 3). (B) Drugs affecting Ca2+ mobilization from internal stores fail to suppress AMPA-accelerated FM1-43 release. Drugs indicated are ryanodine (100 µM; n = 3), dantrolene (50 µM; n = 2), and thapsigargin (2 µM; n = 3), xestospongin (1 µM; n = 3). (C) The endocannabinoid anandamide does not alter conductance in cones. I-V curve from a patch-clamped cone in a flat-mounted salamander retina. Anandamide (100 µM) did not affect the voltage-independent conductance, nor did it change the Ca2+-activation curve, suggesting that anandamide is not the mechanism of positive or negative feedback. (TIF) [file pbio.1001057.s006.tif]
